# Preterm birth, socioeconomic status, and white matter development across childhood

**DOI:** 10.1016/j.dcn.2025.101643

**Published:** 2025-11-08

**Authors:** Katie Mckinnon, Manuel Blesa Cábez, Melissa Thye, Selina Abel, Rebekah Smikle, Jean Skelton, Lorena Jiménez-Sánchez, Kadi Vaher, Gemma Sullivan, Amy Corrigan, Gayle Barclay, Charlotte Jardine, Iona Hamilton, Donna McIntyre, Yu Wei Chua, Ray Amir, Alan J. Quigley, Cheryl Battersby, Athanasios Tsanas, G. David Batty, Rebecca M. Reynolds, Simon R. Cox, Heather C. Whalley, Michael J. Thrippleton, Mark E. Bastin, Hilary Richardson, James P. Boardman

**Affiliations:** a Centre for Reproductive Health, Institute for Regeneration and Repair, University of Edinburgh, UK; b Centre for Clinical Brain Sciences, University of Edinburgh, Edinburgh, UK; c School of Philosophy, Psychology, and Language Sciences, University of Edinburgh, Edinburgh, UK; d Edinburgh Imaging Facility, Royal Infirmary of Edinburgh, Edinburgh, UK; e Department of Radiology, Royal Hospital for Children and Young People, Edinburgh, UK; f Section of Neonatal Medicine, Imperial College London, London, UK; g Usher Institute, Edinburgh Medical School, University of Edinburgh, Edinburgh, UK; h The Alan Turing Institute, London, UK; i Department of Epidemiology and Public Health, University College London, London, UK; j Centre for Cardiovascular Science, University of Edinburgh, Edinburgh, UK; k Lothian Birth Cohorts, Department of Psychology, University of Edinburgh, Edinburgh, UK

**Keywords:** Preterm birth, Socioeconomic status, White matter, Neonate, Child, MRI

## Abstract

Preterm birth and socioeconomic status (SES) are associated with brain development in early life, but the contribution of each over time is uncertain. We examined the effects of gestational age (GA) and SES on white matter microstructure in the neonatal period and at five years.

Participants included preterm and term children. Diffusion MRI was collected at term-equivalent age (n = 153 preterm, n = 90 term [127/243 female]) and from a subset at five years (n = 26 preterm, n = 32 term [22/58 female]). We assessed linear associations of GA, SES (Scottish Index of Multiple Deprivation [SIMD] and maternal education), and GA×SES interactions on fractional anisotropy (FA) using tract-based spatial statistics. We compared the proportion of voxels with significant associations between timepoints.

In preterm neonates, higher GA and higher maternal education, but not SIMD, were associated with higher FA (*p* corrected for family-wise error rate, *p*_*FWER*_ < 0.05). GA-FA associations depended on maternal education and SIMD (β =|0.001–0.005|, *p* < 0.001). At five years, the strength and direction of GA-FA associations depended on SIMD (β =|0.013–0.028|, *p* < 0.001), but not maternal education. In term infants, lower SES was associated with higher FA at the neonatal timepoint only (*p*_*FWER*_ < 0.05).

Preterm birth and SES both shape brain development at birth and continue to do so at five years. The SES measure most strongly associated with FA in preterm infants switches from a family-level (i.e. maternal education) to neighborhood-level (i.e. SIMD) measure between birth and five years, which suggests strategies to mitigate adverse effects of social inequalities on development may require adaptation as children grow.

## Highlights

• Family and neighborhood socioeconomic status associate with fractional anisotropy.
• The nature and direction of these associations are influenced by preterm birth.
• In neonates, fractional anisotropy is most associated with maternal education.
• At five, fractional anisotropy is most associated with neighborhood deprivation.

## Graphical Abstract

**Figure:**
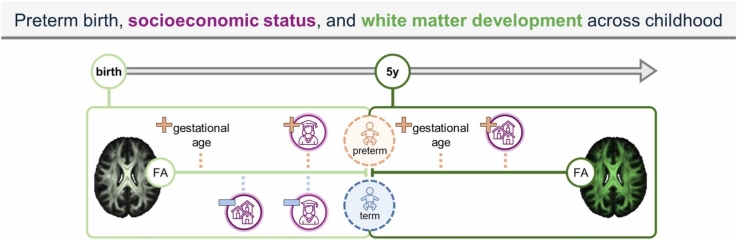

## 1 Introduction

### 1.1 Preterm birth

Preterm birth affects 10 % of children worldwide and is a leading cause of altered white matter development (encephalopathy of prematurity, EoP) and subsequent neurodevelopmental impairment (Ohuma et al., 2023, Johnson and Marlow, 2017). A consistent finding across diffusion magnetic resonance imaging (dMRI) studies is that, compared to term infants, preterm infants have microstructural differences in white matter, including decreased fractional anisotropy (FA) (Barnett et al., 2018, Vaher et al., 2022, Thompson et al., 2019). FA describes the degree of directionality of water diffusion (Pandit et al., 2013). It is widely used to infer microstructural properties of tissues; in the developing brain it is considered a proxy of white matter maturation and a marker of EoP (Kimpton et al., 2021, Volpe, 2009, Inder et al., 2023). FA has proven useful for investigating upstream determinants of brain development; for example, it is associated with postnatal sepsis, histological chorioamnionitis (HCA), bronchopulmonary dysplasia (BPD), necrotizing enterocolitis (NEC), and suboptimal neonatal nutrition (Barnett et al., 2018, Chau et al., 2012, Anblagan et al., 2016, Ball et al., 2010, Lee et al., 2019, Sullivan et al., 2022, Blesa-Cábez et al., 2019), and it associates with neurodevelopmental outcomes (Boardman and Counsell, 2020).

Meta-analytic evidence suggests that variations in brain morphology and microstructure after preterm birth persist (Kelly et al., 2023). For example, studies using dMRI, magnetization transfer ratio, and T1-weighted/T2-weighted signal ratio suggest differences in preterm compared to term children at 4–7 years and in early adolescence (Young et al., 2019, Dodson et al., 2017, Travis et al., 2015, Vandewouw et al., 2019, Thompson et al., 2022). We have built a library of longitudinal brain image data linked to phenotypic data to investigate risk and resilience factors for healthy brain growth in preterm children (Boardman et al., 2020, Boardman et al., 2024).

### 1.2 Socioeconomic status

Socioeconomic status (SES) describes access to financial, educational, social, and health resources, and socioeconomic deprivation is a risk factor for a wide range of health conditions (Bradley and Corwyn, 2002, Kivimäki et al., 2020). In the general population, brain structure, cognition, and educational attainment are associated with SES (Farah, 2017), and in preterm infants SES is an important predictor of neurodevelopmental outcomes (Haider et al., 2025). A systematic review of neuroimaging studies across individuals aged 1–24 years reported associations between SES and structural and dMRI measures (Rakesh and Whittle, 2021). The most consistent finding pertaining to diffusion measures in later childhood is positive higher SES-FA association across some, but not all, white matter tracts (Rakesh and Whittle, 2021, Tooley et al., 2021). However, in children under five years, data are inconsistent with studies reporting positive (Lichtin et al., 2021, Ozernov-Palchik et al., 2019, Thompson et al., 2018, Turesky et al., 2022, Ursache and Noble, 2016), negative (Lean et al., 2022, Sanders et al., 2024), and no SES-FA associations (Lichtin et al., 2021, Turesky et al., 2022, Ursache and Noble, 2016, Lean et al., 2022, Bauer et al., 2019, Takeuchi et al., 2021).

We previously reported that SES and gestational age (GA) are associated with neonatal brain regional volumes, including white matter regions (Mckinnon et al., 2023). Compared to term infants, there is a higher prevalence of socio-economic disadvantage in preterm infants (Thomson et al., 2021, Ruiz et al., 2015), so these children are at risk of the effects of both preterm birth and low SES on brain development.

SES is a multifaceted construct and can be operationalized using neighborhood-level (e.g. postcode-derived deprivation indices) and family-level (e.g. parental education) measures (Pillas et al., 2014, Johnson et al., 2016). These measures, which are only weakly to moderately correlated with one another, capture different aspects of SES and have different impacts on regional brain volumes in preterm infants (Mckinnon et al., 2023). We found more anatomically widespread associations between neonatal regional brain volumes with family- than neighborhood-level SES (Mckinnon et al., 2023). In term infants, neighborhood measures may matter more than parental education as children age (Minh et al., 2017, Gard et al., 2021, Rakesh et al., 2022, Rakesh et al., 2021). The relationships between SES measures, GA, and white matter microstructure across childhood are unknown. Understanding these relationships is needed to target appropriate social support for vulnerable infants.

### 1.3 Aim and hypotheses

We aimed to investigate associations and interactions between SES, preterm birth, and white matter microstructure at term-equivalent age and five years by testing four hypotheses:

1. In preterm infants, lower SES and lower GA associate with lower FA across the white matter skeleton following mutual adjustment at the neonatal and five-year timepoints, and associations of preterm birth are stronger in children from lower SES;
2. SES-FA associations are partially attenuated when adjusting for preterm exposures (sepsis, HCA, BPD, NEC, and low breast milk intake) at the neonatal and five-year timepoints;
3. In term infants, lower SES is associated with lower FA across the white matter skeleton at the neonatal and five-year timepoints;
4. In all participants, associations between neighborhood-level measures of SES and FA in white matter are more widespread at five years than in the neonatal period.

## 2 Methods and materials

### 2.1 Participants

Participants were preterm (< 33 weeks’ GA) and term infants born at the Royal Infirmary Edinburgh, UK, and recruited into the Theirworld Edinburgh Birth Cohort (TEBC). This longitudinal study was designed to investigate the effect of preterm birth on brain development and long-term outcomes (Boardman et al., 2020, Boardman et al., 2024). Recruitment was between 2016 and 2021. Exclusion criteria were major congenital malformation, chromosomal abnormality, and major parenchymal lesions. Ethical approval was from the National Research Ethics Service, South-East Scotland Research Ethics Committee (REC 16/SS/0154), and NHS Lothian Research and Development (2016/0255). Parents provided written informed consent at both timepoints.

At term-equivalent age, 153 preterm and 90 term children had dMRI included (Table 1). Five-year dMRI was included for a subgroup of 26 preterm and 32 term children (Table 1). Data collection is ongoing at the five-year timepoint. Fifty-two children were included at both timepoints (24 preterm and 28 term children). There was no difference in sex distribution between groups (Table 1). Parent-reported child ethnicity did not differ between groups (Table 1) and was representative of Edinburgh (National Records of Scotland, 2020). Fig. S1 gives a flowchart of participants.

**Table 1:** Participant characteristics.

		**Neonatal timepoint**		**Five-year timepoint**	
**Characteristic**		**Preterm [n = 153]**	**Term [n = 90]**	**Preterm [n = 26]**	**Term [n = 32]**
Median birth gestation/weeks + day, (IQR)		30 + 0 (26 +5–33 +1)	39 + 5 (38 +2–41 +1)	30 + 0 (27 +6–32 +1)	39 + 5 (38 +4–40 +6)
Median birthweight/grams, (IQR)		1320 (765–1875)	3460 (2920–4000)	1420 (1103–1737)	3380 (2868–3892)
Sex	Female	87/153 (56.9 %)	40/90 (44.4 %)	11/26 (42.3 %)	11/32 (34.4 %)
	Male	66/153 (43.1 %)	50/90 (55.6 %)	15/26 (57.7 %)	21/32 (65.6 %)
Median age at MRI at each timepoint Neonatal: corrected gestation/weeks + day, (IQR) Five-year: months (IQR)		40 + 4 (38 +5–42 +3)	42 + 0 (40 +3–43 +4)	61.7 (60.6–62.8)	61.6 (60.4–62.8)
SIMD rank, median (IQR)		4086 (325–6966)	5360 (2323–6967)	4510 (1570–6929)	5471 (3251–6931)
Maternal education^a^	No qualifications	5/153 (3.3 %)	0/90 (0.0 %)	0/26 (0 %)	0/32 (0 %)
	Basic high school qualification [1–4 passes]	8/153 (5.2 %)	2/90 (2.2 %)	1/26 (3.8 %)	0/32 (0 %)
	Basic high school qualification [> 4 passes]	10/153 (6.5 %)	3/90 (3.3 %)	0/26 (0 %)	1/32 (3.1 %)
	Advanced high school qualification	11/153 (7.2 %)	2/90 (2.2 %)	1/26 (3.8 %)	0/32 (0 %)
	College qualification	43/153 (28.1 %)	9/90 (10.0 %)	7/26 (26.9 %)	2/32 (6.3 %)
	University undergraduate degree	43/153 (28.1 %)	39/90 (43.3 %)	10/26 (38.5 %)	15/32 (46.9 %)
	University postgraduate degree	33/153 (21.6 %)	35/90 (38.9 %)	7/26 (26.9 %)	14/32 (43.8 %)
Child ethnicity	Any white background	133/153 (86.9 %)	79/87 (90.8 %)	24/26 (92.3 %)	30/32 (93.8 %)
	Mixed background^b^	9/153 (5.9 %)	4/87 (4.6 %)	1/26 (3.8 %)	2/32 (6.3 %)
	Other ethnic group^c^	11/153 (7.2 %)	3/87 (3.4 %)	1/26 (3.8 %)	0/32 (0 %)
Preterm exposures^d^	BPD	33/151 (21.9 %)	NA	2/24 (8.3 %)	NA
	HCA	47/140 (33.6 %)	NA	8/25 (32 %)	NA
	Low breast milk intake	75/151 (50.0 %)	NA	6/25 (24.0 %)	NA
	NEC	4/153 (2.6 %)	NA	0/26 (0 %)	NA
	Sepsis	33/153 (21.6 %)	NA	3/26 (11.5 %)	NA
Sum of preterm exposures, median (IQR)		1 (0–3)	NA	1 (0–2)	NA

IQR = inter-quartile range.

a Maternal education levels are defined in eMethods.

b Mixed background includes for preterm children in neonatal period: Black Caribbean and Pakistani (n = 1), Japanese Brazilian and German (n = 1), Scottish and Kurdish (n = 1), White and Asian (n = 4), White and Black African (n = 1), White and Egyptian (n = 1). For term children in neonatal period: Armenian and German (n = 1), White and Black Caribbean (n = 2), White and Middle Eastern (n = 1). For preterm children at five years: White and Black African (n = 1). For term children at five years: Armenian and German (n = 1), Chinese and White (n = 1).

c Other ethnic group includes for preterm children in neonatal period: African (n = 2), Arab (n = 2), White Bulgarian/Turkish (n = 2), Indian (n = 1), Iraqi (n = 1), Pakistani (n = 3). For term children in neonatal period: Bangladeshi (n = 1), Malaysian (n = 1), Pakistani (n = 1). For preterm children at five years: African (n = 1).

d BPD = requirement for respiratory support and/or supplemental oxygen after 36 weeks’ corrected gestation. HCA = chorioamnionitis on histological analysis of the placenta by histopathologist. Low breast milk intake = defined as < 75 % days during neonatal unit stay with exclusive maternal or donor breast milk. NEC = includes both medical (seven days nil by mouth) or surgical management. Sepsis = positive blood culture and/or physician decision to treat with five days of antibiotics including both early- and late-onset sepsis.

### 2.2 Imaging

#### 2.2.1 Magnetic resonance imaging acquisition

At term-equivalent age, MRI scans were performed according to published protocol (Boardman et al., 2020). A Siemens MAGNETOM Prisma 3 T MRI clinical scanner (Siemens Healthcare, Erlangen, Germany) and 16-channel phased-array pediatric head receive coil were used to acquire a multishell axial dMRI scan (16 × b = 0 s/mm^2^, 3 × b = 200 s/mm^2^, 6 × b = 500 s/mm^2^, 64 × b = 750 s/mm^2^, 64 × b = 2500 s/mm^2^) with optimal angular coverage (see eMethods for full protocol). Infants were fed, wrapped, and slept naturally. Flexible earplugs and neonatal earmuffs (MiniMuffs, Natus) were used for acoustic protection. Infants were monitored throughout, and scans were supervised by a doctor or nurse trained in neonatal resuscitation.

At five years, the same MRI scanner with a 32-channel phased-array head receive coil was used to acquire a 3D T1-weighted MPRAGE scan (voxel size = 1 mm isotropic) and a multishell axial dMRI scan (15 × b = 0 s/mm^2^, 3 × b = 200 s/mm^2^, 6 × b = 500 s/mm^2^, 64 × b = 1000 s/mm^2^, 64 × b = 2000 s/mm^2^) with optimal angular coverage (see eMethods for full protocol) (Boardman et al., 2024). Children were introduced to the scanner using a child-friendly booklet and mock-scan session with simulated MRI scanner. Children wore in-ear headphones (Nordic Neuro Lab, Bergen, Norway) to minimize scanner noise and enable listening to movies of their choice. During the scan, a researcher stood at the foot of the bore to monitor the child. If the child moved visibly, the researcher reminded them to stay still.

All structural images were reviewed by a radiologist with experience in pediatric MRI (AJQ) and conventional findings from the neonatal timepoint are reported (Sullivan et al., 2024). No participants in this study had major parenchymal lesions (Fig. S1).

#### 2.2.2 Data pre-processing

At term-equivalent age, raw images were visually inspected before processing and low-quality images discarded (Fig. S1). For each participant, the two dMRI acquisitions were concatenated and denoised using Marchenko-Pastur principal component analysis-based algorithm with MRtrix3 (Tournier et al., 2019, Veraart et al., 2016); eddy current, head movement and echo planar imaging (EPI) geometric distortions were corrected using outlier replacement and slice-to-volume registration (Andersson et al., 2017, Andersson et al., 2016, Andersson et al., 2003, Andersson and Sotiropoulos, 2016) using FMRIB Software Library (FSL) (Smith et al., 2004); bias field inhomogeneity correction was performed by calculating the bias field of mean b0 volume and applying correction to volumes (Tustison et al., 2010) using MRtrix3.

At five years, pre-processing was similar. Images were visually inspected, b = 200 s/mm^2^ removed, dMRI data denoised (Tournier et al., 2019, Veraart et al., 2016) and Gibbs unringing performed using MRtrix3 (Kellner et al., 2016). Eddy current, head movement and EPI geometric distortions were corrected using outlier replacement and slice-to-volume registration incorporating susceptibility-by-movement interactions with eddy currents (Andersson et al., 2017, Andersson et al., 2016, Andersson et al., 2003, Andersson and Sotiropoulos, 2016, Andersson et al., 2018); bias field inhomogeneity correction was performed by calculating the bias field of mean b0 volume and applying correction to volumes (Tustison et al., 2010)*.* Finally, images were resampled to 1.5 mm isotropic voxels.

Diffusion tensor imaging (DTI) maps were calculated using dMRI processed images to obtain FA. A DTI model was fitted in each voxel using weighted least-squares method DTIFIT in FSL, using only b = 750 s/mm^2^ shell for neonates and b = 1000 s/mm^2^ for five years.

#### 2.2.3 Voxel-wise analyses

Voxel-wise statistical analyses of FA data were carried out using Tract-Based Spatial Statistics (TBSS) (Smith et al., 2006) and Randomise in FSL (Smith et al., 2004). Separately for the two timepoints, all participants’ FA data were transformed into a common space, i.e. the most representative participant for each timepoint, identified as the participant requiring minimum transformation. This used affine and non-linear registration (Andersson et al., 2007a, Andersson et al., 2007b), with additional initial rigid registration for term-equivalent age (Ball et al., 2010). In addition, the selected participant was non-rigidly registered to age-specific templates for each timepoint (Avants et al., 2011, Warrington et al., 2020, Uus et al., 2021, Jenkinson et al., 2012). This transformation was concatenated to the previous and applied to each participant. Next, the mean FA image was created separately for timepoints and thinned to create a mean FA skeleton representing the centers of all tracts common to the group, with threshold FA 0.15 for term-equivalent age, and 0.2 for five years. Each participant’s aligned FA data was projected onto their corresponding skeleton and the resulting data fed into voxel-wise cross-participant statistics.

### 2.3 Variables and covariates

We assessed SES using two measures. Neighborhood-level SES was defined using the Scottish Index of Multiple Deprivation rank (SIMD, 2016) (Scottish National Statistics, 2016), derived from family postcode at birth, analyzed as a continuous measure from 1 (most deprived) to 6976 (least deprived). The index incorporates neighborhood indicators of income, employment, education, health, access to services, crime, and housing (Scottish National Statistics, 2016). For visualization of interaction effects, these were combined into two groups, namely SIMD quintiles 1–4 and SIMD quintile 5. Family-level SES was operationalized using ordered categories of maternal education, obtained from parent-reported highest educational qualification at recruitment (see supplementary eMethods for categories). For visualization of interaction effects, these were combined into two groups, namely maternal non-university education and university education.

GA was derived from medical records, as a continuous variable (Preacher, 2015, Fisher et al., 2020). Age at MRI scan was included as a continuous measure in all analyses for each timepoint. At term-equivalent age this was corrected GA in weeks, and at five years this was chronological age in years.

Sex was derived from medical records and included because there are sex differences in white matter development through childhood (Kumpulainen et al., 2023). Preterm exposures associated with FA were combined into a single measure with a total score of 0–5 (Barnett et al., 2018, Conole et al., 2023), counting the following exposures for every infant: BPD, HCA, low breast milk intake, NEC, and sepsis (Barnett et al., 2018, Chau et al., 2012, Anblagan et al., 2016, Ball et al., 2010, Lee et al., 2019, Sullivan et al., 2022, Blesa-Cábez et al., 2019). Preterm exposure definitions are detailed in eMethods.

### 2.4 Statistical analyses

#### 2.4.1 Tract-based spatial statistics

Voxel-wise statistical analysis of FA data was performed using Randomise in FSL (Smith et al., 2004, Winkler et al., 2014). In timepoint-specific analyses, multivariable linear regression models assessed the relationship between FA and GA, SES (maternal education and SIMD separately), and a multiplication interaction between SES and GA (removed if no significant association seen), with sex, age at scan, and an intercept as covariates. For preterm infants, analyses were then adjusted for preterm exposures by inclusion as an additional covariate. Each analysis included family-wise error correction for multiple comparisons and threshold-free cluster enhancement, with statistical significance of *p* corrected for family-wise error rate (*p*_*FWER*_) < 0.05 (Winkler et al., 2016, Smith and Nichols, 2009).

Statistical analyses were preregistered (Mckinnon et al., 2024). In the whole cohort, in the neonatal period we found positive and negative associations between GA and FA, and small FA regions associated with an interaction between maternal education and GA (Tables S1–2, Figs. S2–3). The interaction was such that higher GA associated with lower FA for children of mothers with no educational qualifications, but higher GA associated with higher FA for children of mothers with any educational qualifications (Fig. S3). At the five-year timepoint, we found small regions with a positive association between GA and FA. GA and SIMD interacted, such that higher GA associated with lower FA for children in families living in the least deprived neighborhoods (Fig. S2 and S4). These observations suggested that relationships between GA, SES and FA differ by gestational category. Therefore, we undertook the voxel-wise statistical analyses separately for the preterm and term infants. Subgroup analyses of term infants were not preregistered. Details of each regression model are in Table S3.

#### 2.4.2 Comparison of SES effects on FA between birth and five years

We counted the number of voxels where there was a statistically significant association (*p*_*FWER*_<0.05) between FA and GA, maternal education, SIMD, or a multiplication interaction between GA and each SES measure. We counted the total voxels tested at each timepoint to account for volume differences. We compared the proportion of the white matter skeleton with significant associations with each SES measure and each interaction between timepoints using McNemar tests in R. This analysis was modified from the preregistered analysis plan to account for the additional subgroup analyses (Mckinnon et al., 2024).

## 3 Results

### 3.1 Associations between gestational age, socioeconomic status, and fractional anisotropy in preterm infants

#### 3.1.1 Neonatal timepoint

**Fig. 1:**
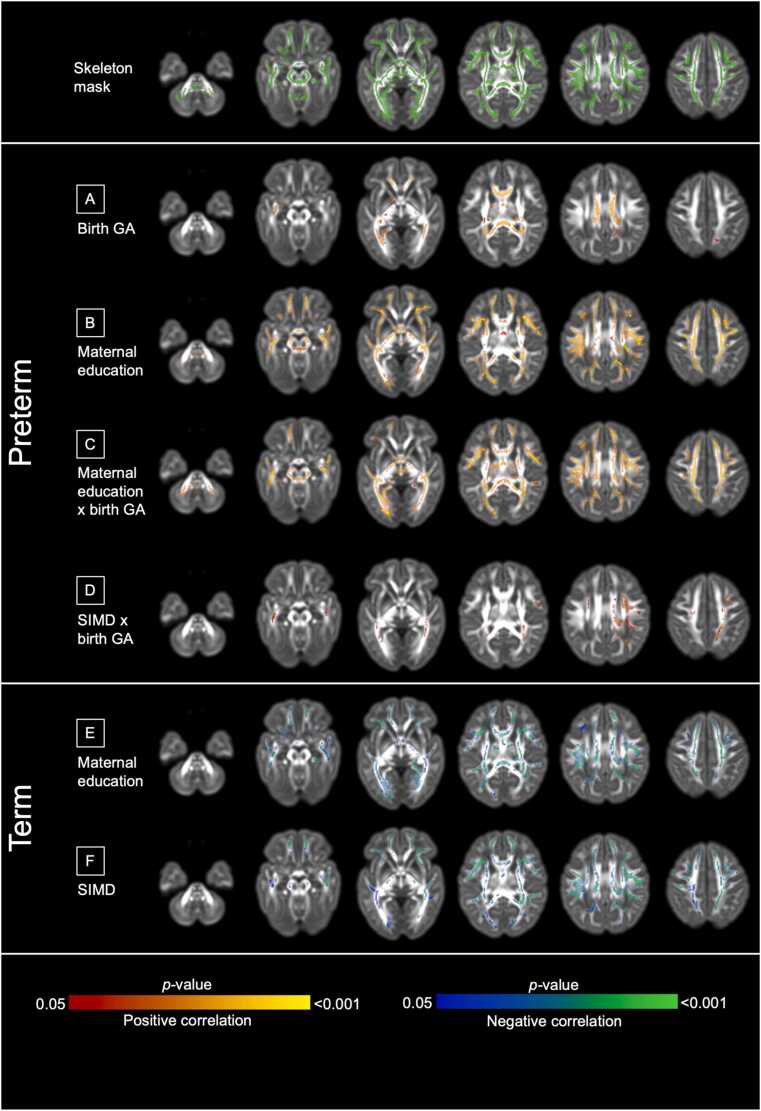
*Voxels where fractional anisotropy associations were found in the neonatal period.* In preterm infants (n = 153), models were mutually adjusted for GA, SES (maternal education and SIMD separately), GA × SES interaction, sex, and age at MRI. A: GA, B: maternal education, C: maternal education × GA, D: SIMD × GA. In term infants (n = 90), models were adjusted for sex and age at MRI. E: maternal education, F: SIMD. Results are reported after 5000 permutations, *p*-values corrected using threshold-free cluster enhancement and family-wise error correction with a significance level of *p* < 0.05. For visualization: anatomic left is on the left side of the image. Red-yellow indicates a positive association, blue indicates a negative association. Results are displayed overlaid on the dHCP neonatal template (Uus et al., 2021) for the neonatal timepoint.

Among preterm infants at term-equivalent age (n = 153), infants born at higher GA had higher FA in the corpus callosum, posterior corona radiata, and cingulum bundles, after adjustment for sex and age at scan, irrespective of SES measure included in the model. Fig. 1A shows results adjusted for maternal education and similar results were seen in the model adjusted for SIMD.

**Fig. 2:**
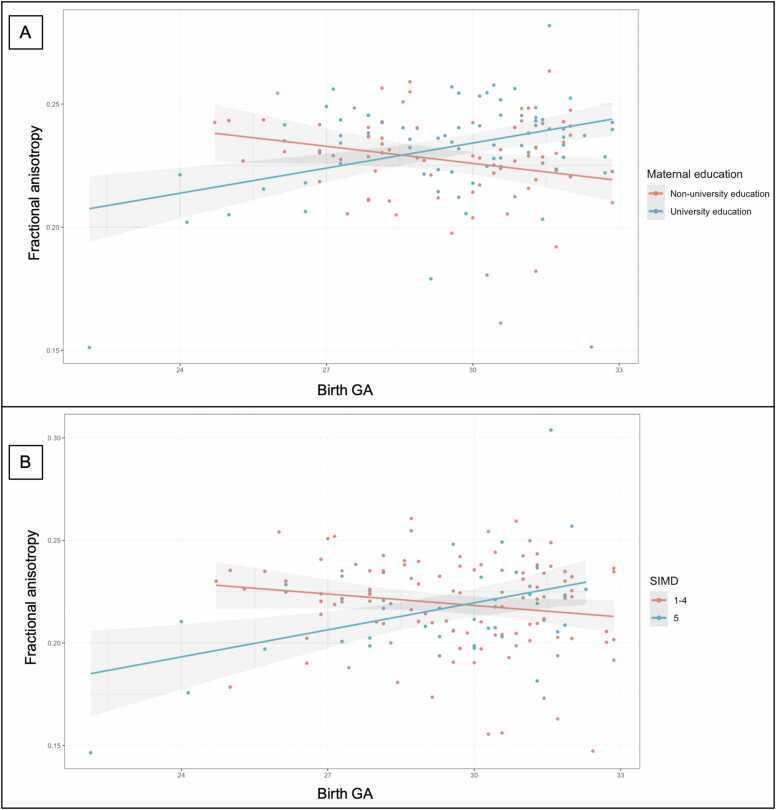
*Interaction effects between gestational age and socioeconomic status on fractional anisotropy at term-equivalent age in preterm infants.* A: Interaction effect between GA and maternal education on FA. B: Interaction effect between GA and SIMD on FA. Term-equivalent age in preterm infants (n = 153). For each individual, mean FA was extracted from voxels where there are significant interaction effects. Interaction effects all *p* < 0.001. Maternal education analyses were performed using maternal education as seven categories (see eMethods), visualized as linear regression lines for two groups for ease of interpretation, namely maternal non-university education (no qualifications, basic or advanced high school qualifications, or college qualification, n = 77) and university education (undergraduate or postgraduate degrees, n = 76). SIMD analyses were performed using SIMD rank as a continuous measure, visualized as two groups for ease of interpretation, namely SIMD quintiles 1–4 (n = 109) and SIMD quintile 5 (n = 44). A version of Fig. 2B with all SIMD quintiles visualized can be seen in Fig. S5.

Higher maternal education was associated with higher FA across the white matter (Fig. 1B), and there were anatomically widespread GA × maternal education interaction effects on FA (Fig. 1C). Specifically, higher GA associated with higher FA for neonates of mothers with university education (n = 76), whereas higher GA associated with lower FA for infants of mothers without university education (n = 77) (β=0.003 and β=-0.002 respectively, *p* < 0.001) (Fig. 2A).

There were no independent associations between SIMD and FA at term-equivalent age. However, there was significant interaction between GA and SIMD with FA such that higher GA associated with higher FA in the corona radiata and superior longitudinal fasciculus, for children born into the least deprived neighborhoods (Fig. 1D). The interaction is illustrated in Fig. 2B and Fig. S5, which shows higher GA is associated with higher FA for children whose families are living in less deprived neighborhoods (SIMD quintile 5, n = 44), whereas higher GA associated with lower FA for children whose families are living in the more deprived neighborhoods (SIMD quintiles 1–4, n = 109) (β = 0.005 and β = -0.001 respectively, *p* < 0.001).

**Fig. 3:**
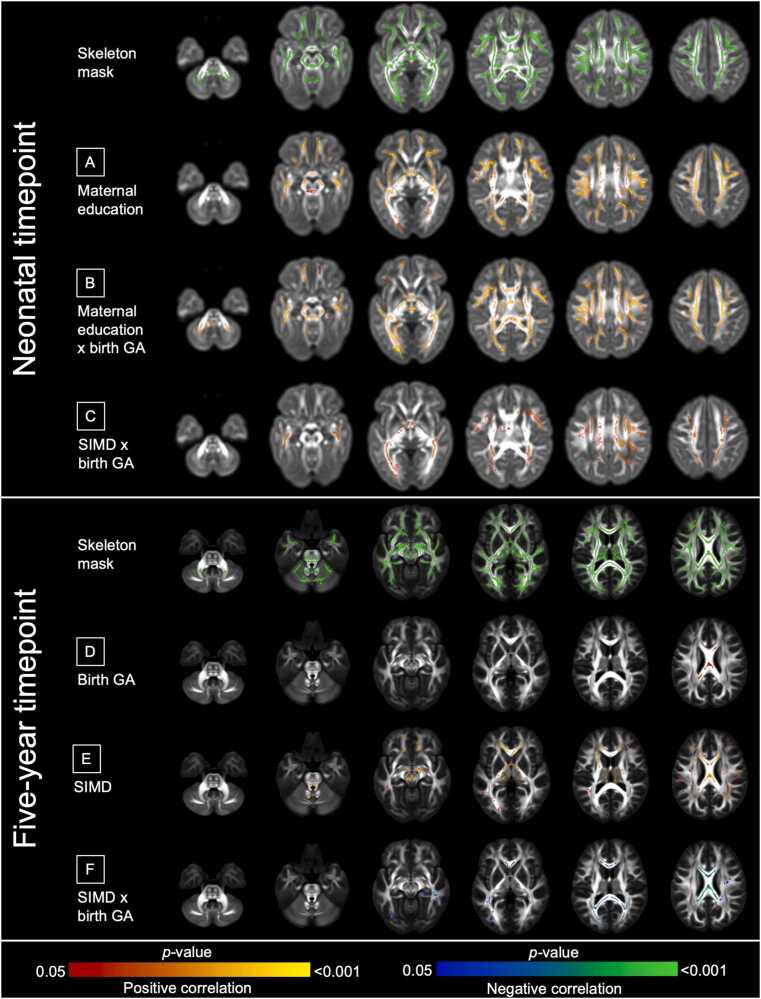
*Associations between gestational age, socioeconomic status, and fractional anisotropy in preterm infants after adjustment for co-exposures of preterm birth.* Voxels where FA associations were found in preterm infants at the neonatal timepoint (n = 153) and at the five-year timepoint (n = 26) after adjustment for co-exposures. Models were mutually adjusted for GA, SES (maternal education and SIMD separately), GA × SES interaction, sex, age at MRI, and sum of preterm exposures (BPD, HCA, low breast milk intake, NEC, and sepsis). In the neonatal period - A: Maternal education, B: maternal education × GA, C: SIMD × GA. At five years - D: GA, E: SIMD, F: SIMD × GA. Results are reported after 5000 permutations, p-values corrected using threshold-free cluster enhancement and family-wise error correction with a significance level of *p* < 0.05. For visualization: anatomic left is on the left side of the image. Red-yellow indicates a positive association, blue indicates a negative association. Overlaid on the developing Human Connectome Project (dHCP) neonatal template (Uus et al., 2021) for the neonatal timepoint, and on the FSL HCP1065 (Human Connectome Project) adult template (Warrington et al., 2020) for the five-year timepoint.

Associations between GA and FA were no longer significant in models adjusted for co-morbidities of preterm birth (Table S4). However, associations between FA and maternal education, and the interaction between GA and maternal education or SIMD, were minimally affected (Fig. 3A–C, Table S4).

#### 3.1.2 Five-year timepoint

**Fig. 4:**
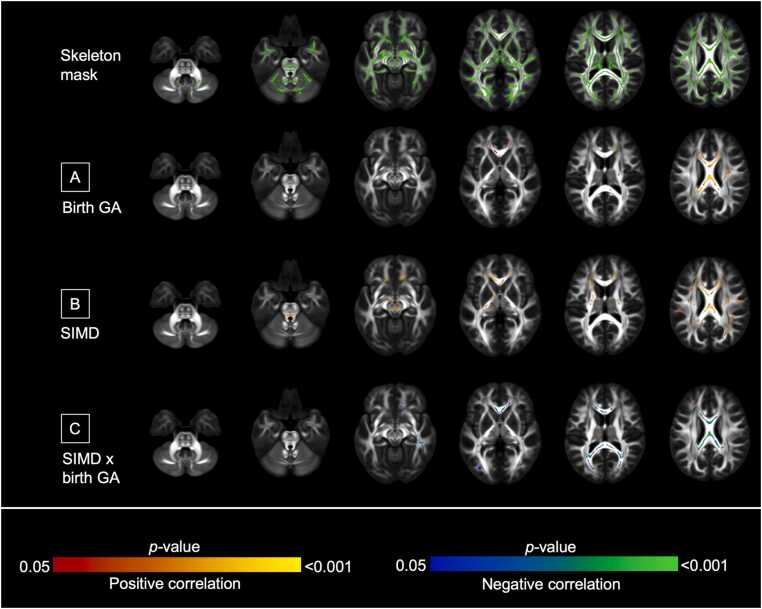
*Voxels where fractional anisotropy associations were found at five years of age in preterm infants.* Voxels where FA associations were found in preterm infants at the five-year timepoint (n = 26). Models were mutually adjusted for GA, SES (maternal education and SIMD separately), GA × SES interaction, sex, age at MRI. A: GA, B: SIMD, C: SIMD × GA. Results are reported after 5000 permutations, *p*-values corrected using threshold-free cluster enhancement and family-wise error correction with a significance level of *p* < 0.05. For visualization: anatomic left is on the left side of the image. Red-yellow indicates a positive association, blue indicates a negative association. Overlaid on the FSL HCP1065 adult template (Warrington et al., 2020) for the five-year timepoint.

In preterm infants at five years (n = 26), there were associations between higher GA and higher FA in the corpus callosum (Fig. 4A), and between higher SIMD and higher FA in the corpus callosum, internal capsule, and brainstem (Fig. 4B). There were associations in the corpus callosum between the interaction of GA × SIMD on FA, such that higher GA associated with higher FA in children living in more deprived neighborhoods, whereas higher GA associated with lower FA for children in less deprived neighborhoods (β = 0.028 and β = -0.013 respectively, *p* < 0.001, Fig. 4C and Fig. S6). These remained significant after adjustment for perinatal exposures, although the proportion of the white matter skeleton associated with GA reduced (5.6 % [5816/104,030 voxels] to 0.9 % [959/104,030 voxels]) (Fig. 3D–F, Table S4). There were no associations between FA and maternal education, or an interaction between GA and maternal education, at five years.

### 3.2 Associations between socioeconomic status and fractional anisotropy in term infants

#### 3.2.1 Neonatal timepoint

In term infants (n = 90), higher SES associated with lower FA across the white matter (Fig. 1E–F). Both maternal education (Fig. 1E) and SIMD (Fig. 1F) showed similarly anatomically widespread associations in term neonates, in contrast to preterm infants when maternal education has more widespread associations than neighborhood deprivation.

#### 3.2.2 Five-year timepoint

In term infants at five years (n = 32), there were no associations between either SES measure and FA.

### 3.3 Differences in socioeconomic status-fractional anisotropy associations between the neonatal period and five years

In preterm infants, there were differences over time in the proportion of the white matter skeleton containing significant voxel-wise associations between SES and SES × GA interactions with FA (*p* < 0.001). At the neonatal timepoint, maternal education (57.5 %, 16,890/29,354 voxels) and GA × maternal education (53 %, 15,544/29,354) were associated with FA in a higher proportion of the white matter skeleton compared to SIMD (0 %) or an interaction between SIMD and GA (8.6 %, 2526/29,354). However, at five years, SIMD (14.8 %, 15,381/104,030) or the interaction between GA and SIMD (6.7 %, 7008/104,030) had the most widespread associations with FA (Table 2).

**Table 2:** Number of significant voxels where FA associates with SES, GA, or an interaction between SES and GA.

**Group**	**Variable**	**Number (proportion) of significant voxels at the neonatal timepoint**	**Number (proportion) of significant voxels at the five-year timepoint**
Preterm	GA (in model with maternal education)	3934 (13.4 %)	0
	GA (in model with SIMD)	4014 (13.7 %)	5816 (5.6 %)
	Maternal education	16,890 (57.5 %)	0
	Maternal education × GA	15,544 (53 %)	0
	SIMD	0	15,381 (14.8 %)
	SIMD × GA	2526 (8.6 %)	7008 (6.7 %)
Term	Maternal education	12,941 (44.1 %)	0
	SIMD	12,945 (44.1 %)	0

Number of significant voxels (*p* corrected for family-wise error rate (*p*_*FWER*_) < 0.05) where FA associates with SES, GA, or an interaction between SES and GA. Total number of voxels at neonatal timepoint = 29,354, and at five years of age = 104,030.

In term infants at both timepoints there were similar proportions of white matter showing SES-FA associations with each SES measure. At the neonatal timepoint, maternal education and SIMD were similarly associated with FA (44.1 % [12,941/29,354] and 44.1 % [12,945/29,354] voxels respectively). At five years, there were no associations between maternal education or SIMD with FA (Table 2).

## 4 Discussion

### 4.1 Key findings

In a contemporary birth cohort enriched for prematurity, GA and SES were associated with FA across the white matter skeleton during the neonatal period and at five years. The extent and direction of these associations depend on gestational category (preterm versus term) and SES measure.

Among preterm infants, the observation that higher GA is associated with higher FA within the white matter is consistent across many studies (Inder et al., 2023). Here we further show that GA-FA associations reduced after adjustment for preterm adverse co-exposures (sepsis, HCA, BPD, NEC, and low breast milk intake). This corroborates growing evidence indicating that it is not low GA *per se* that drives the development of encephalopathy of prematurity and adverse outcomes, but rather, it is adverse medical and environmental risks experienced by preterm infants, such as immune dysregulation and sub-optimal nutrition, that play a key role in atypical brain development of preterm infants (Boardman and Counsell, 2020, Twilhaar et al., 2018, Boardman et al., 2007). In addition, we found striking associations between SES and FA that were more widespread across the neonatal WM skeleton than those attributable to low GA. Of SES measures tested, maternal education was more strongly associated with neonatal brain development than neighborhood deprivation revealing a consistent pattern that for children from higher SES families, higher GA is associated with higher FA but in children from lower SES families higher GA associates with lower FA. In sum, the relationship between degree of prematurity and neonatal white matter development is modified by maternal education level and neighborhood deprivation.

There were differences in the direction of SES-FA associations between preterm and term infants. In preterm infants, higher maternal education was associated with higher FA, whereas in term infants, higher maternal education and living in a less deprived neighborhood associated with lower FA. This unexpected finding could be due to the greater susceptibility of the preterm brain to socioeconomic influences. Preterm infants may have an accelerated maturation of white matter, manifesting as higher FA, which is not seen in term infants. SIMD and maternal education appear to have similar effects in term infants, but among preterm infants, maternal education has a stronger role in the neonatal period. This could be due to the family-level measure better capturing adversities in the antenatal and early neonatal periods.

In preterm infants, when adjusting for preterm co-exposures and co-morbidities, associations between SES and SES × GA and FA are minimally changed, both in the magnitude of association and distribution of nominally significant voxels. This suggests that associations between SES and white matter microstructure are independent of these early life exposures. This may be because preterm co-exposures have such a strong influence on FA that this is independent of SES. However, interventions beyond the medical care of preterm infants are likely needed to effectively address the influence of SES on brain development.

In five-year-old preterm children, FA associated with SIMD and an interaction between GA and SIMD, indicating that for those with lower SES, higher GA was associated with higher FA, but there was a negative association between GA and FA in the higher SES group. In contrast, in term children at five years, SES measures were not associated with FA.

The finding of different directions of SES-FA associations at the two timepoints is consistent with the protracted development hypothesis (Tooley et al., 2021); this could explain the negative SES-FA association in term infants and the negative GA-FA association for preterm infants from lower SES backgrounds in the neonatal period. This hypothesis describes how higher SES is associated with more protracted structural and functional development, with slower initial development followed by rapid development in later childhood and adolescence (Tooley et al., 2021). Although reviews in older children have suggested a positive SES-FA association (Rakesh and Whittle, 2021, Tooley et al., 2021), the variability in findings in younger children (Lichtin et al., 2021, Ozernov-Palchik et al., 2019, Turesky et al., 2022, Ursache and Noble, 2016, Lean et al., 2022, Sanders et al., 2024, Bauer et al., 2019, Takeuchi et al., 2021) may be explained by a more complex relationship with age. These complex relationships are also consistent with previous morphometric similarity network analyses that demonstrate dysmaturation such that some networks have accelerated maturation while others are delayed (Galdi et al., 2020). Given the varying nature and direction of SES-FA associations seen here, imaging techniques allowing more inference about microstructural development than FA, and those combining different microstructural indices are likely to be informative in future studies of SES-brain interactions in early life.

Looking across timepoints in preterm infants, the SES measure most strongly associated with FA changed over time, from maternal education in the neonatal period to neighborhood deprivation at five years. This is consistent with previous work in this cohort showing that family-level SES (i.e. maternal education) was associated with more regional brain volumes than neighborhood-level SES (i.e. SIMD) at term-equivalent age (Mckinnon et al., 2023), and findings that SIMD was associated with developmental outcomes in later infancy, including a preference to viewing social stimuli (Dean et al., 2021) and emotional regulation and cortisol responses (Ginnell et al., 2022). There is further evidence in the wider literature that neighborhood measures may matter more than parental education as children age (Minh et al., 2017, Gard et al., 2021, Rakesh et al., 2022, Rakesh et al., 2021). This may be due to family-level SES measures capturing the in utero and perinatal exposures, whereas neighborhood-level SES measures that capture wider socioeconomic circumstances become more influential over time.

These data are broadly consistent with previous findings from this cohort describing the relationship between SES and volumetric and cortical structural measurements on neonatal brain MRI (Mckinnon et al., 2023). SES correlates with early brain development in term and preterm infants, and the way SES is operationalized and the gestational category of the infant are both important. This study offers additional insight via diffusion MRI and the longitudinal design. Microstructure appears to be more sensitive to perinatal exposures that macrostructure; factors such as BPD and nutrition have more observable impacts on measures of microstructure than macrostructure, suggesting that network formation is a susceptible target at this stage of development.

### 4.2 Strengths and limitations

This is a rare cohort study of preterm and term children including MRI at two timepoints linked to detailed characterization of medical and social data. The preterm children did not have major parenchymal brain injuries, so are representative of most survivors of modern intensive care practices (Boardman et al., 2020, Boardman et al., 2024, Sullivan et al., 2024). We included two measures of SES, allowing comparison of how dimensions of SES vary in their association with FA. We adjusted for preterm exposures, testing the robustness of GA-FA and SES-FA associations in the context of other disease processes affecting brain dysmaturation.

FA is an established marker of encephalopathy of prematurity that is sensitive to upstream determinants of brain development and is associated with neurocognitive outcomes (Inder et al., 2023, Parikh, 2016), and we used an established processing pipeline that is unbiased to particular tracts. There are some limitations to the use of FA as a metric of white matter microstructure; FA differences can represent differences in a number of different microstructural elements in a voxel (e.g. fiber density, myelination), but because of the complexity of the white matter with crossing fibers, FA differences could be due to disproportionate development or degeneration of a fiber bundle (Figley et al., 2022). However, there are studies showing association between FA and histological myelination (Lazari and Lipp, 2021), lending support to the use of FA as a proxy for white matter integrity.

There are some limitations. Although the cohort is comparable to neonatal populations in high-income, highly educated, majority-white settings, further work is needed to determine whether the social patterns we observed are generalizable to settings with different socioeconomic or ethnicity profiles. We studied two SES measures, but did not have data for all that could be relevant, such as household income, nor could we study the impact of social mobility on FA in this study group (Cerutti et al., 2021, McLoughlin et al., 2022, Castagné et al., 2016). There was a difference in age at MRI scan between the preterm and term infants in the neonatal period, but we adjusted for this within regression models that included both groups.

The 5-year sample is smaller than the neonatal sample because data collection at 5 years is ongoing. Nevertheless, based on previous sensitivity analyses of simulated data we estimate that in the smallest groupwise comparison we performed TBSS should identify differences in FA in 60–70 % of affected voxels (Ball et al., 2013). Longitudinal analyses using dMRI metrics and behavioral testing are planned to better understand how SES and preterm birth might affect the rate of cerebral development and functional abilities over childhood (Boardman et al., 2020, Boardman et al., 2024).

### 4.3 Conclusions

Family- and neighborhood-level measures of SES are associated with white matter microstructure in the developing brain across childhood. However, the nature and direction of these associations are influenced by preterm birth and specific SES measures. In preterm infants, low GA and SES are associated with white matter microstructure in the newborn period and at five years, but the SES measure most closely associated with FA changes from maternal education in the neonatal period to neighborhood deprivation at five years. Preterm birth appears to expose a susceptibility of the developing brain to social disadvantage that persists through childhood. Strategies to mitigate the adverse effects of social disadvantage on child development may require a personalized approach that takes account of gestational age at birth and the timing of intervention, focusing on family-level interventions in very early life with increasing focus on neighborhood-level interventions as children grow up.

## CRediT authorship contribution statement

**Katie Mckinnon:** Writing – review & editing, Writing – original draft, Visualization, Resources, Methodology, Investigation, Formal analysis, Data curation, Conceptualization. **Manuel Blesa Cábez:** Writing – review & editing, Visualization, Software, Methodology, Investigation, Formal analysis, Data curation, Conceptualization. **Melissa Thye:** Writing – review & editing, Visualization, Software, Methodology, Investigation, Formal analysis, Data curation, Conceptualization. **Selina Abel:** Writing – review & editing, Resources, Investigation. **Rebekah Smikle:** Writing – review & editing, Investigation. **Jean Skelton:** Writing – review & editing, Investigation. **Lorena Jiménez-Sánchez:** Writing – review & editing, Resources, Investigation. **Kadi Vaher:** Writing – review & editing, Investigation, Formal analysis. **Gemma Sullivan:** Writing – review & editing, Investigation. **Amy Corrigan:** Writing – review & editing, Project administration, Data curation. **Gayle Barclay:** Writing – review & editing, Resources, Investigation. **Charlotte Jardine:** Writing – review & editing, Resources, Investigation. **Iona Hamilton:** Writing – review & editing, Resources, Investigation. **Donna McIntyre:** Writing – review & editing, Resources, Investigation. **Yu Wei Chua:** Writing – review & editing, Resources, Investigation, Data curation. **Ray Amir:** Writing – review & editing, Resources, Investigation, Data curation. **Quigley Alan J:** Writing – review & editing, Resources, Investigation. **Cheryl Battersby:** Writing – review & editing, Funding acquisition. **Athanasios Tsanas:** Writing – review & editing, Funding acquisition. **G David Batty:** Writing – review & editing, Funding acquisition. **Reynolds Rebecca M:** Writing – review & editing, Funding acquisition. **Cox Simon R:** Writing – review & editing, Methodology, Funding acquisition. **Whalley Heather C:** Writing – review & editing, Methodology, Funding acquisition. **Thrippleton Michael J:** Writing – review & editing, Resources, Methodology. **Bastin Mark E:** Writing – review & editing, Software, Resources, Funding acquisition. **Hilary Richardson:** Writing – review & editing, Writing – original draft, Visualization, Supervision, Resources, Project administration, Methodology, Funding acquisition, Formal analysis, Conceptualization. **Boardman James P:** Writing – review & editing, Writing – original draft, Visualization, Supervision, Resources, Methodology, Funding acquisition, Formal analysis, Conceptualization.

## Data statement

Deidentified participant data is available to researchers whose proposed use of the data has been approved as part of a data access agreement:

https://reproductive-health.ed.ac.uk/theirworld-edinburgh-birth-cohort-tebc/for-researchers/data-access-and-collaboration

## Disclosures

All authors declare that they have no competing interests.

## Declaration of Competing Interest

The authors declare that they have no known competing financial interests or personal relationships that could have appeared to influence the work reported in this paper.

## Data Availability

Deidentified participant data is available to researchers whose proposed use of the data has been approved as part of a data access agreement: https//reproductive-health.ed.ac.uk/theirworld-edinburgh-birth-cohort-tebc/for-researchers/data-access-and-collaboration.
